# Prediction of competing endogenous RNA coexpression network as prognostic markers in AML

**DOI:** 10.18632/aging.101985

**Published:** 2019-05-31

**Authors:** Jun-Dan Wang, Hong-Sheng Zhou, Xi-Xiang Tu, Yi He, Qi-Fa Liu, Quentin Liu, Zi-Jie Long

**Affiliations:** 1 Department of Hematology, The Third Affiliated Hospital, Sun Yat-sen University, Guangzhou 510630, China; 2 Institute of Hematology, Sun Yat-sen University, Guangzhou 510630, China; 3 Department of Hematology, Nanfang Hospital, Southern Medical University, Guangzhou 510000, China; 4 Institute of Cancer Stem Cell, Dalian Medical University, Dalian 116000, China; * Equal contribution

**Keywords:** ceRNA coexpression network, AML, prognostic markers, aging, age-related diseases

## Abstract

Recently, competing endogenous RNAs (ceRNAs) hypothesis has gained a great interest in the study of molecular biological mechanisms of cancer occurrence and progression. However, studies on leukemia are limited, and there is still a lack of comprehensive analysis of lncRNA-miRNA-mRNA ceRNA regulatory network of AML based on high-throughput sequencing and large-scale sample size. We obtained RNA-Seq data and compared the expression profiles between 407 normal whole blood (GTEx) and 151 bone marrows of AML (TCGA). The similarity between two sets of genes with trait in the network was analyzed by weighted correlation network analysis (WGCNA). MiRcode, starBase, miRTarBase, miRDB and TargetScan was used to predict interactions between lncRNAs, miRNAs and target mRNAs. At last, we identified 108 lncRNAs, 10 miRNAs and 8 mRNAs to construct a lncRNA-miRNA-mRNA ceRNA network, which might act as prognostic biomarkers of AML. Among the network, a survival model with 8 target mRNAs (HOXA9+INSR+KRIT1+MYB+SPRY2+UBE2V1+WEE1+ZNF711) was set up by univariate and multivariate cox proportional hazard regression analysis, of which the AUC was 0.831, indicating its sensitivity and specificity in AML prognostic prediction. CeRNA networks could provide further insight into the study on gene regulation and AML prognosis.

## INTRODUCTION

Acute myeloid leukemia (AML), characterized by abnormal proliferation and differentiation of myeloid progenitor cells, is an aggressive hematological malignancy. Hematopoietic transformation leads to modification in numbers of key transcriptional targets during myelopoiesis. Alterations occur in genes with important roles in regulation of hematopoietic progenitors, contribute to hematological pathogenesis, and could represent attractive targets for AML treatment [1]. In recent years, numbers of reports of the competing endogenous RNAs (ceRNAs) network has emerged in the study of AML development and therapy [2, 3].

The hypothesis of ceRNAs states the pool of long non-coding RNAs (lncRNAs), pseudogenes, circular RNAs (circRNAs) and messenger RNAs (mRNAs), compete and bind to microRNAs (miRNAs), regulating their activity [4, 5]. Among the ceRNA, lncRNAs have attracted much attention, as accumulating evidence has revealed that lncRNAs are involved in a wide range of biological processes. MiRNAs regulate the expression of the target genes by binding to the miRNA response elements (MREs) on the target mRNAs. And, lncRNAs act as molecular sponges to attract miRNAs, contributing to various human diseases process [6]. At present, the ceRNA hypothesis has been proven to be implicated in the development of different kinds of tumors, such as liver, gastric, breast, colon, pancreatic and bladder cancer.

In chronic myeloid leukemia (CML), lncRNA SNHG5 promoted imatinib resistance via acting as a ceRNA against miR-205-5p [7]. LncRNA UCA1 was also identified as an important modulator of MDR1 to promote imatinib resistance through completely binding miR-16 [8]. In AML, lncRNA NEAT1 modulated cell proliferation and apoptosis by regulating miR-23a-3p/SMC1A [9]. LncRNA UCA1 contributed to the chemoresistance, through activating glycolysis by the miR-125a/HK2 pathway [10]. In addition, aberrant upregulation of CCAT1 was detected in French-American-British (FAB) M4 and M5 subtypes of AML patients. CCAT1 repressed monocytic differentiation and promoted cell growth by up-regulating c-Myc via its ceRNA activity on miR-155 [11]. Sen et al. explored the major cross-talking edges of ceRNA networks in CML and AML utilizing patient sample data, which shed light on progression and prognosis of leukemia [12]. Therefore, studies have showed that the lncRNA-miRNA-mRNA ceRNA regulatory network is implicated in the leukemia development. However, studies on leukemia are limited, and there is still a lack of comprehensive analysis of lncRNAs, miRNAs and mRNAs related to AML based on high-throughput sequencing and large-scale sample size.

In this study, we obtained RNA-Seq data and compared the expression profiles between 151 bone marrows (BMs) of AML (The Cancer Genome Atlas, TCGA) [13] and 407 normal whole blood (Genotype-Tissue Expression, GTEx) [14, 15]. Following, mRNAs and lncRNAs between the normal samples and AML patients were applied to weighted correlation network analysis (WGCNA) to enrich modules which were most related with AML [16]. And, miRNA database was used to predict target mRNA. Finally, we identified 108 lncRNAs, 10 miRNAs and 8 mRNAs to construct a lncRNA-miRNA-mRNA ceRNA network. Among the network, a survival model with 8 target mRNAs (HOXA9+INSR+KRIT1+MYB+SPRY2+UBE2V1+WEE1+ZNF711) was set up for predicting AML prognosis.

## RESULTS

### Different gene expression from data between TCGA and GTEx is analyzed

**Figure 1:**
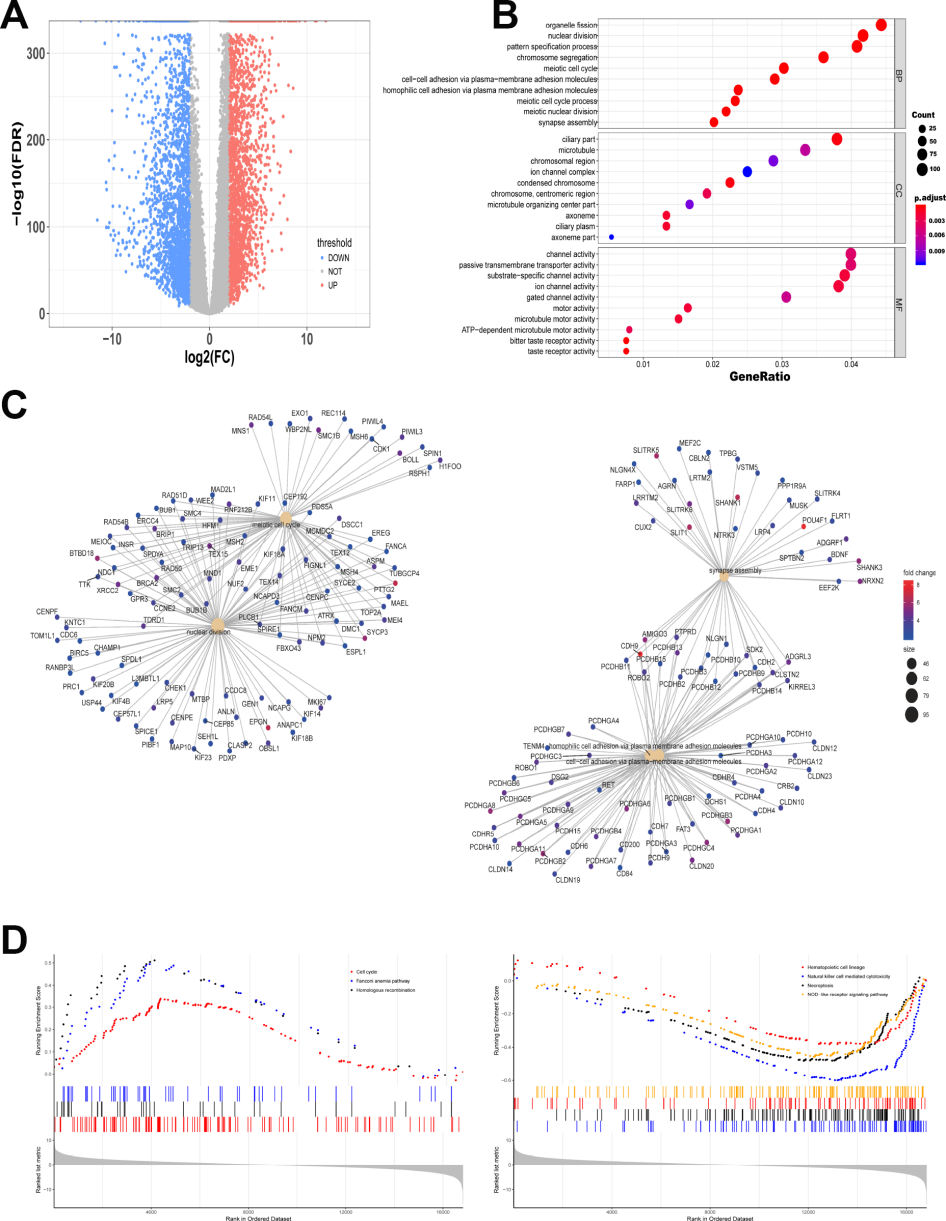
**Different gene expression from data between TCGA and GTEx is analyzed.** (**A**) Volcano map of significantly different expression of mRNAs. Red spots represent up-regulated genes, and blue spots represent down-regulated genes. (**B**) Information from up-regulated genes was applied to GO analysis in BP, CC and MF. (**C**) Gene symbols and interaction of the significantly up-regulated mRNAs in BP were shown. (**D**) KEGG-GSEA was applied for signaling pathway analysis.

The expression levels of RNAs in 151 bone marrow samples with AML and 407 normal whole blood samples were explored. The clinicopathological and molecular characteristics of AML patients were shown in Table 1 and Table 2. All gene read counts were normalized to the trimmed mean of M values (TMM) by edgeR. We found that 2667 significantly up-regulated mRNAs and 2456 down-regulated mRNAs were identified. Figure 1A showed the distribution of all the significantly different expressed mRNAs on the two dimensions of -log10 (false discovery rate, FDR) and log2 (fold change, FC) through a volcano map. The gene modules in the network are often enriched with specific functions, which are of biological significance. To test the biological function of the identified genes, information from differentially expressed genes was applied to Gene Ontology (GO) analysis. Up-regulated mRNAs were enriched in organelle fission, nuclear division and pattern specification process in biological process (BP) (Figure 1B). Figure 1C showed the gene symbols and their interactions in BP of up-regulated mRNAs. Moreover, cell cycle, fanconi anemia pathway and homologous recombination related genes were upregulated while hematopoietic cell lineage, natural killer cell mediated cytotoxicity, necroptosis and NOD-like receptor signaling pathways were downregulated by Kyoto Encyclopedia of Genes and Genomes (KEGG)-Gene Set Enrichment Analysis (GSEA) (Figure 1D).

**Table 1:** The clinicopathological characteristics of AML patients.

	**Alive(n=54)**	**Dead(n=97)**	**Total(n=151)**
**Gender**			
FEMALE	24(44.4%)	44(45.4%)	68(45.0%)
MALE	30(55.6%)	53(54.6%)	83 (55.0%)
**Age**			
Mean(SD)	47.4(14.2)	58(15.9)	54.2(16.1)
Median[MIN, MAX]	50[21,74]	62[21,88]	56[21,88]
**FAB classification**			
M0	5(9.3%)	10(10.3%)	15(9.9%)
M1	11(20.4%)	24(24.7%)	35(23.2%)
M2	14(25.9%)	24(24.7%)	38(25.2 %)
M3	11(20.4%)	4(4.1%)	15(9.9%)
M4	8(14.8%)	21(21.6%)	29(19.2%)
M5	5(9.3%)	10(10.3%)	15(9.9%)
M6		2(2.1%)	2(1.3%)
M7		1(1.0%)	1(0.7%)
Not Classified		1(1.0%)	1(0.7%)

**Table 2:** The cytogenetic risk, immunophenotype and mutation of AML patients.

**Cytogenetic Risk Group — no.(%)**		
Favorable	31	20.5
Intermediate	82	54.3
Poor	36	23.8
Missing data	2	1.3
**Immunophenotype — no.(%)**		
CD33+	124	82.1
CD34+	99	65.6
CD117+	134	88.7
**Mutation — no.(%)**		
DNMT3A	18	12.6
FLT3	45	30.6
NPM1	33	22.0
RAS	8	5.3
IDH1	26	17.2

Abbreviations: DNMT3A data were available among 143 patients (data from Simple Nucleotide Variation-Masked Somatic Mutation of TCGA). The data of FLT3, NPM1, RAS and IDH1 were available among 147, 150, 150 and 151 patients respectively.

### WGCNA is applied to analyze gene modules

**Figure 2:**
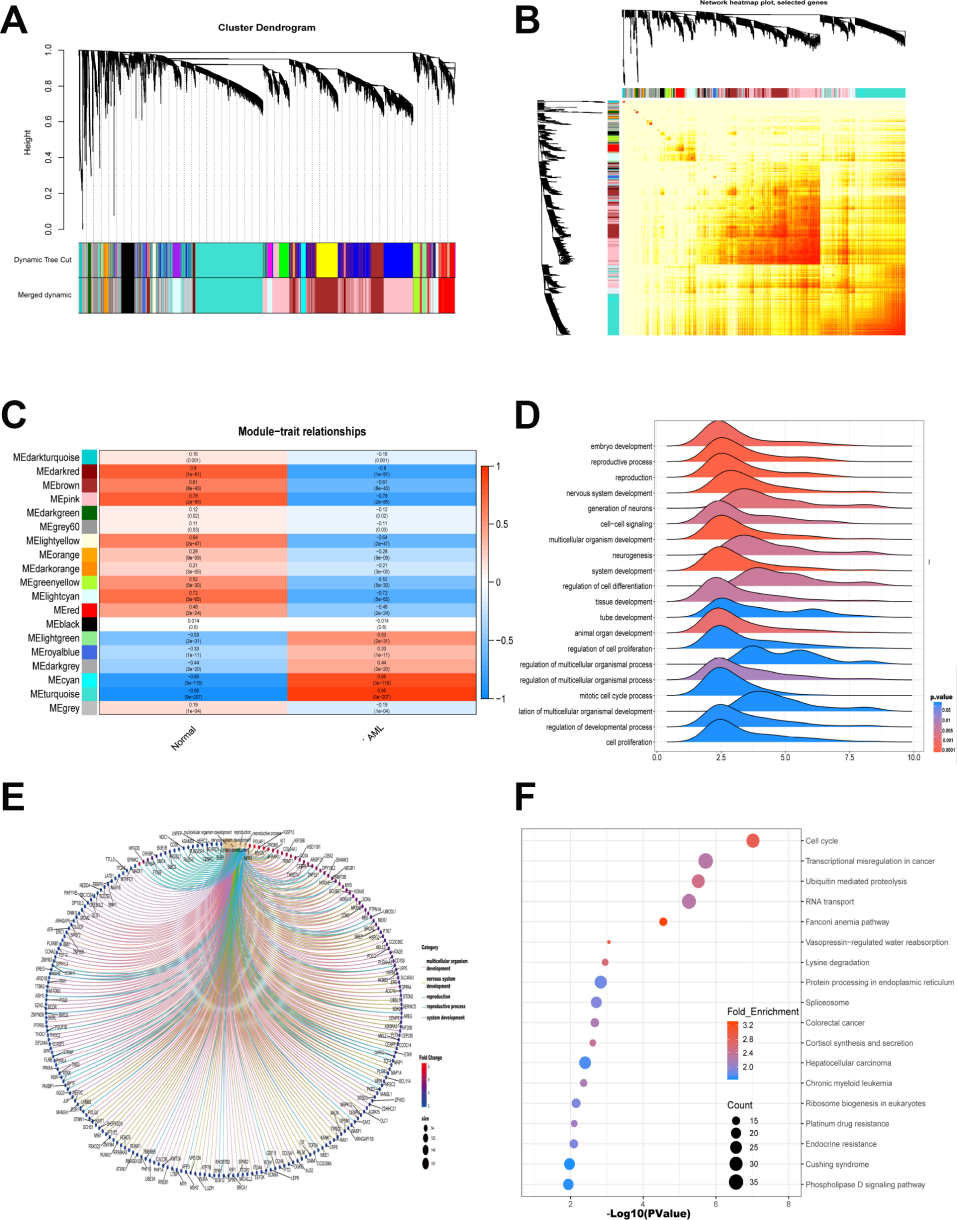
**WGCNA is applied to analyze gene modules.** (**A**) Cluster dendrogram of the coexpression network modules was produced based on topological overlap in the mRNAs. (**B**) Heatmap plot of topological overlap in the gene network was shown. (**C**) The relation of genes in modules between AML and normal samples was investigated. (**D**–**E**) GO-GSEA displayed the gene symbols and gene interaction in cyan module and turquoise module. (**F**) KEGG analysis was used to investigate the pathway enrichment in cyan module and turquoise module.

Gene modules were analyzed using the WGCNA among the first 40% mRNAs by variance comparison. As shown in Figure 2A, softpower 7 and module size cut-off 25 were chosen as the threshold to identify coexpressed gene modules. 19 gene color modules were identified and the heatmap plot of topological overlap matrix (TOM) was shown in Figure 2B. Then, genes in the 19 color modules were continuously used to analyze the module-trait (AML and normal) coexpression similarity and adjacency. Cyan module and turquoise module showed high relationship with AML, which included 1659 mRNAs (Figure 2C). These 1659 mRNAs were further used to GO-GSEA to display the gene enrichment, gene symbols and their interactions in BP, as shown in Figure 2D and 2E. The genes were most related to embryo development, reproductive process and reproduction. In addition, genes were highly enriched in cell cycle, transcriptional misregulation in cancer, ubiquitin mediated proteolysis and RNA transport by KEGG analysis (Figure 2F).

### LncRNAs modules are analyzed by WGCNA

**Figure 3:**
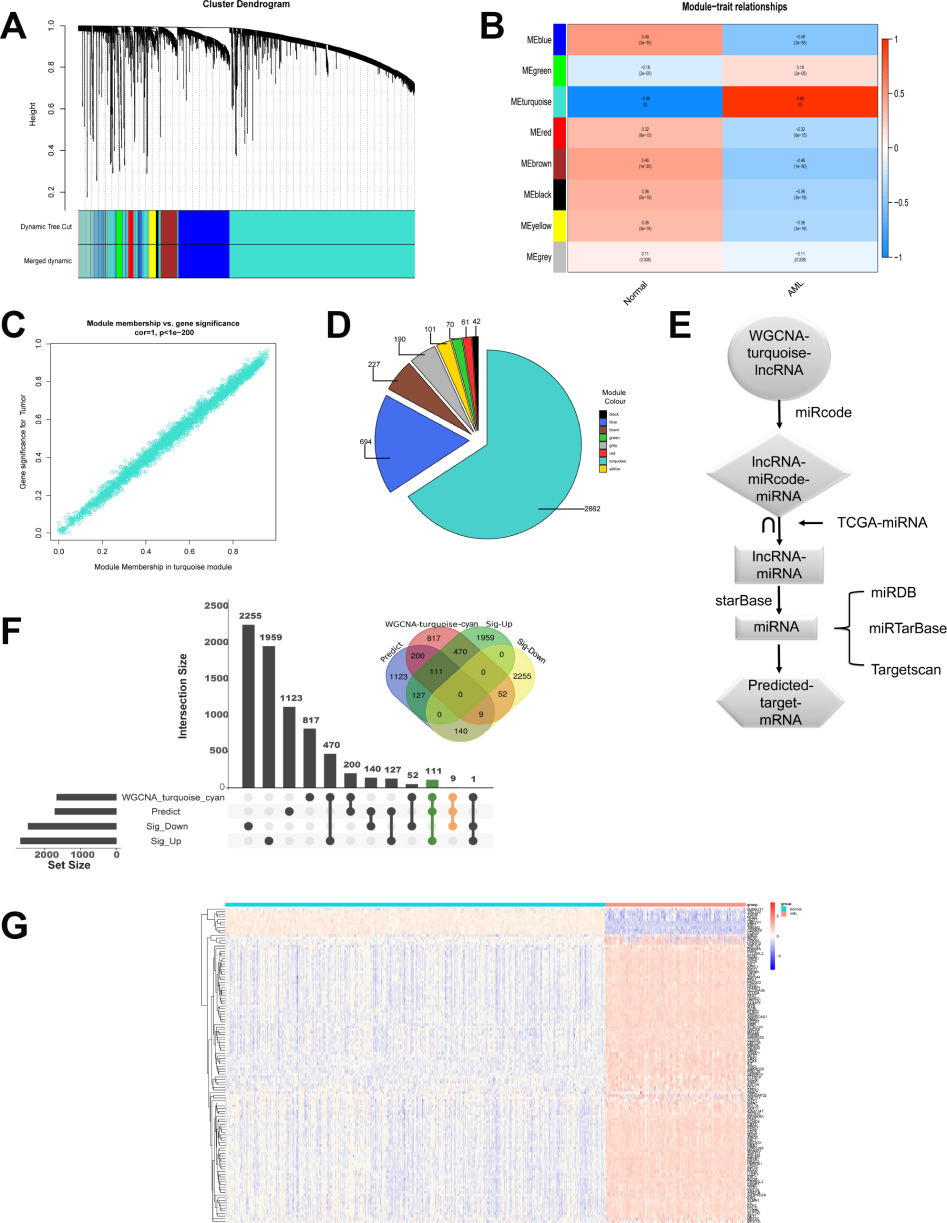
**LncRNAs modules are analyzed by WGCNA.** (**A**) Cluster dendrogram of the coexpression network modules was produced based on topological overlap in the lncRNAs. (**B**) The relation of lncRNAs in modules between AML and normal samples was investigated. (**C**) Turquoise module showed highest relationship with AML. (**D**) The number of lncRNAs in every module was shown. (**E**) Flow chart exhibited the process of predicting target mRNAs. (**F**) Overlapped target mRNAs were analyzed by the predicted target mRNAs, WGCNA-cyan-turquoise mRNAs, and the significantly up-regulated mRNAs and down-regulated mRNAs. (**G**) The expression of 120 selected target genes was displayed by heatmap.

Next, we continued to investigate coexpression network of lncRNAs. LncRNA modules were analyzed by WGCNA among the first 60% lncRNAs by variance comparison. As shown in Figure 3A, softpower 6 was chosen as the threshold and we identified 8 coexpressed lncRNA modules. Correlation analysis showed that turquoise module displayed highest relationship with AML (Figure 3B and 3C; r=0.98). The numbers of lncRNAs in every module were shown in Figure 3D. The turquoise module contained the highest numbers (2662) of lncRNAs. We then used miRcode to predict the miRNAs sponged by 2662 lncRNAs to obtain lncRNAs-miRcode-miRNAs relationship. Meanwhile, we used TCGA miRNA-Seq to analyze the first 400 miRNA with highest expression. Then the overlapped miRNAs between 400 miRNAs and lncRNAs-miRcode-miRNAs (155) were selected to obtain lncRNAs-miRNAs (47). We further explored and obtained 1710 predicted target mRNAs by starBase, miRDB, miRTarBase and Targetscan dataset, which might be bound by 47 miRNAs (Figure 3E). Importantly, as shown in Figure 3F, we chose the overlapped target mRNAs by analyzing the predicted target mRNAs (1710), WGCNA-turquoise-cyan mRNAs (1659), as well as the significant differentially up-regulated mRNAs (2667) and down-regulated mRNAs (2456) by edgeR. Lastly, we got 111 up-regulated mRNAs and 9 down-regulated mRNAs (Supplementary Table 1). The expression of these 120 genes in 558 samples was shown in Figure 3G by heatmap.

### Cox regression analysis is conducted to clarify the patients’ survival

**Figure 4:**
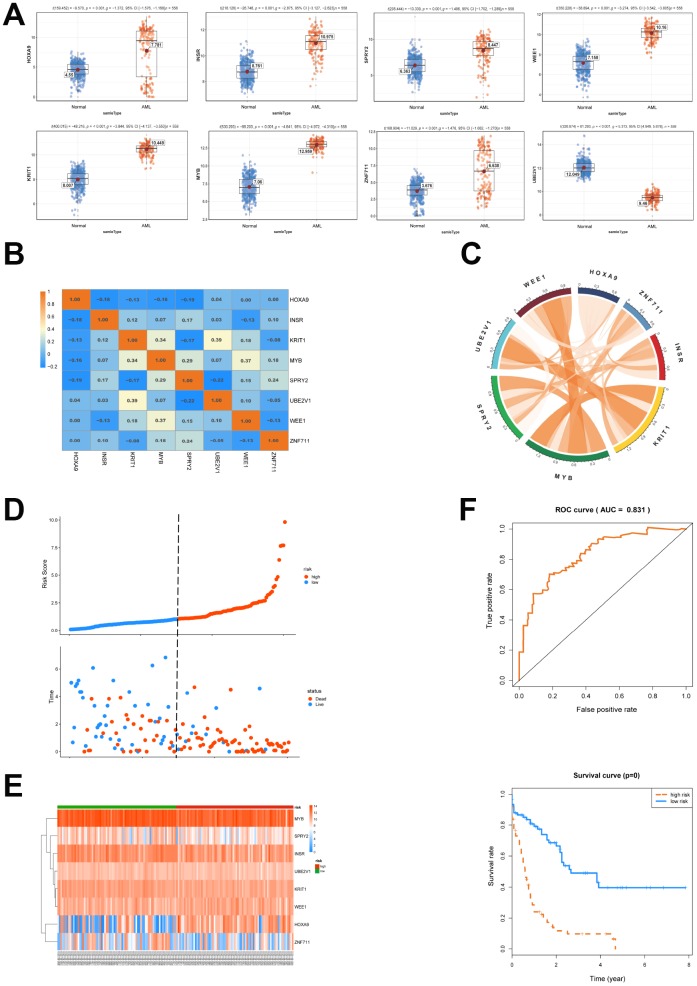
**Survival analysis of the 8 genes is conducted.** (**A**) The expression of 8 selected genes between AML and normal samples was shown. (**B**, **C**) The expression relationship of the 8 genes was displayed. (**D**) AML patients were classified into predicted low and high risk groups according to the multivariate cox proportional hazard regression analysis. (**E**) The expression heatmap of the 8 genes in high risk or low risk group was shown. (**F**) ROC and Kaplan-Meier survival analysis of the 8- genes model was performed.

Next, a univariate cox proportional hazard regression analysis was conducted to clarify the association of the expression levels of 120 genes with overall survival (OS). 22 genes were obtained by the threshold of p value <0.05 and gene ID <15000 (NCBI). The above mentioned 22 genes were brought into further multivariate cox proportional hazard regression analysis (Table 3). We then set up a survival model for 3-year OS with 8 genes: HOXA9+INSR+KRIT1+MYB+SPRY2+UBE2V1+WEE1+ZNF711. We showed that HOXA9, INSR, KRIT1, MYB, SPRY2, WEE1 and ZNF711 were up-regulated while UBE2V1 was down-regulated in AML patients (Figure 4A). The correlationship of each gene in the 8-genes model was shown in Figure 4B and 4C. The patients from TCGA were classified into predicted low and high risk groups according to the multivariate cox score result in Figure 4D. Furthermore, the expression heatmap of the 8 genes in high risk or low risk group was shown in Figure 4E. We then estimated the accuracy of the 8-genes signature on predicting survival. Kaplan-Meier survival curves showed that patients with predicted high risk (n=75) had significantly shorter OS than those with low risk (n=76, p=0.00, Figure 4F). Receiver operating characteristic (ROC) analysis to compare the sensitivity and specificity of the survival prediction of our models was performed. TCGA dataset revealed that the area under receiver operating characteristic curve (AUC) of the 8-genes signature was 0.831. Previous reports showed that gene mutation was correlated with the prognosis of AML [17]. Thus, we divided the patients into groups according to gene mutations and we found that the 8-genes signature worked well in DNMT3A, FLT3 or RAS mutation, as well as NPM1 wildtype patient subgroups (Supplementary Figure 1).

**Table 3:** Multivariate cox proportional hazard regression analysis of 22 genes.

**Gene**	**Univariate**		**Multivariate**	
	**HR(95%CI)**	**P**	**HR**	**P**
INSR	0.603 (0.47–0.77)	0.0001	0.759	0.040 *
MYB	0.618 (0.48–0.79)	0.0002	0.625	0.022 *
HOXA9	1.112 (1.05–1.18)	0.0002	1.097	0.002 **
HOXA10	1.124 (1.05–1.2)	0.0004		
KRIT1	0.455 (0.29–0.71)	0.0004	0.678	0.131
RREB1	0.364 (0.21–0.64)	0.0005		
REV3L	0.559 (0.39–0.81)	0.0018		
RAB5B	0.543 (0.37–0.8)	0.0023		
CLOCK	0.605 (0.43–0.85)	0.0038		
MEIS1	1.087 (1.02–1.15)	0.0063		
PTPN14	0.876 (0.8–0.96)	0.0069		
CDK6	0.753 (0.61–0.93)	0.0102		
MEF2C	1.230 (1.05–1.44)	0.0110		
KIT	0.869 (0.78–0.97)	0.0118		
SPRY2	0.888 (0.81–0.98)	0.0152	0.892	0.074
ZNF460	0.749 (0.59–0.95)	0.0168		
ZNF711	0.930 (0.87–0.99)	0.0229	0.940	0.100
WEE1	1.394 (1.04–1.87)	0.0280	1.757	0.002**
MEST	0.896 (0.81–0.99)	0.0344		
RCN2	0.657 (0.44–0.97)	0.0369		
UBE2V1	0.591 (0.36–0.97)	0.0373	0.500	0.009**
EREG	1.061 (1–1.12)	0.0454		

### A lncRNA-miRNA-mRNA ceRNA network is constructed

**Figure 5:**
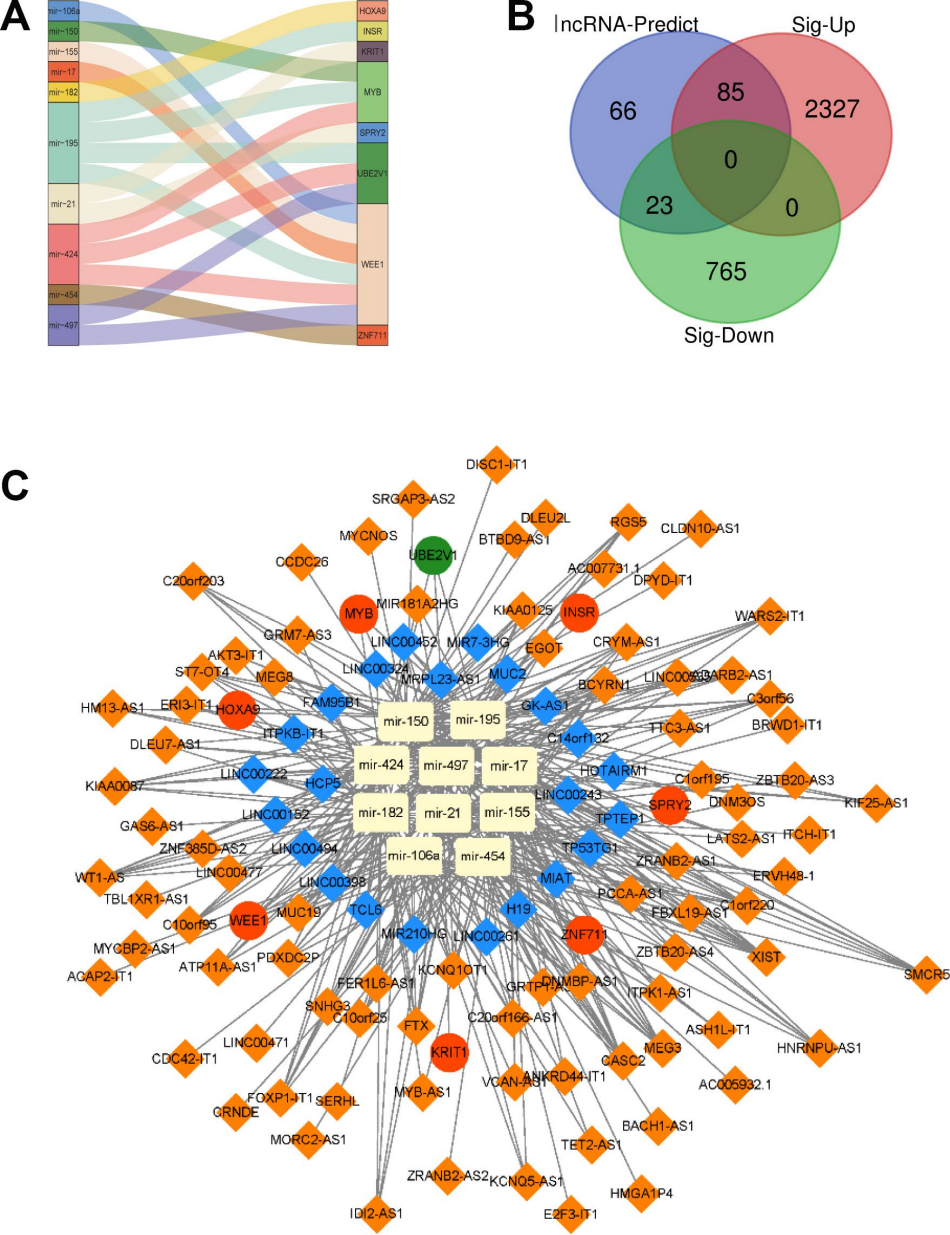
**A lncRNA-miRNA-mRNA ceRNA network is constructed.** (**A**) The relationship between the 8 target genes and their corresponding miRNA was shown. (**B**) Overlapped lncRNAs were analyzed by the predicted lncRNAs, significantly up-regulated lncRNAs and down-regulated lncRNAs. (**C**) A lncRNA-miRNA-mRNA ceRNA network was constructed by 108 lncRNAs, 10 miRNAs and 8 mRNAs for AML prognosis.

In the following step, we showed the relation between the 8 target genes and their corresponding miRNAs. We found that miR-106a, miR-150, miR-155, miR-17, miR-182, miR-195, miR-21, miR-424, miR-454 and miR-497 could target the 8 mRNAs respectively. For example, miR-195 targeted INSR, MYB, WEE1 and UBE2V1, while miR-106a, miR-155, miR-17, miR-195, miR-424 and miR-497 regulated WEE1 (Figure 5A). Since TCGA and GTEx also provided the data of lncRNAs, the differentially expressed lncRNAs were also analyzed by edgeR. 2412 up-regulated lncRNAs and 788 down-regulated lncRNAs were identified. Then these 3200 lncRNAs were overlapped with the lncRNAs (174) predicted from 10 miRNAs, and we got 108 different expressed 108 lncRNAs (Figure 5B). At last, a lncRNA-miRNA-mRNA ceRNA network was constructed by 108 lncRNAs, 10 miRNAs and 8 mRNAs, as shown in Figure 5C.

## DISCUSSION

Important advance in ceRNA network research developed rapidly, suggesting that the involvement of ceRNA network in human diseases, especially tumors, could be far more prevalent. The disruption of the equilibrium of ceRNA network was critical for tumorigenesis. Thus, understanding the intricate interplay among diverse ceRNA network will lead to significant insight into gene regulatory networks and have implications in cancer treatment [2]. Here, we identified 108 lncRNAs, 10 miRNAs and 8 mRNAs to construct a lncRNA-miRNA-mRNA ceRNA network by database. Given the fact there was no large-scale of public RNA-Seq database or studies with normal BMs, further validation of normal BMs by large cohorts are needed.

In lung cancer, lncRNA BARD1 9'L, transcribed from an alternative promoter in intron 9 of the BARD1 gene and shared part of the 3'UTR with the protein coding BARD1 mRNAs, counteracted the effect of miR-203 and miR-101, to promote tumor development [18]. LncRNA HOTAIR, functioned as a ceRNA, sponging miR-331-3p to derepress HER2, which was correlated with advanced gastric cancers [19]. SNHG7, whose high expression was correlated with poor prognosis, acted as a target of miR-34a to increase GALNT7 level and regulate PI3K/Akt/mTOR pathway in colorectal cancer progression [20]. Thus, ceRNA network displayed essential role in cancer progress and provided potent targets for cancer therapy.

Importantly, lncRNA-miRNA-mRNA ceRNA network can be predicted for disease prognosis. For example, in the study of RNA-Seq data of breast cancer from TCGA, a lncRNA-miRNA-mRNA ceRNA network was established, which comprised of 8 miRNAs, 48 lncRNAs, and 10 mRNAs. A multivariate cox regression analysis demonstrated that 4 of those lncRNAs (ADAMTS9-AS1, LINC00536, AL391421.1 and LINC00491) had significant prognostic value [21]. In pancreatic cancer, 11 lncRNAs, A2M-AS1, DLEU2, LINC01133, LINC00675, MIR155HG, SLC25A25-AS1, LINC01857, LOC642852 (LINC00205), ITGB2-AS1, TSPOAP1-AS1 and PSMB8-AS1 were identified and validated on a pancreatic ductal adenocarcinoma expression dataset. Moreover, A2M-AS1, LINC01133, LINC00205 and TSPOAP1-AS1 were identified as prognostic biomarkers [22]. In glioblastoma multiforme, lung cancer, ovarian cancer and prostate cancer, based on the networks, only a fraction of sponge lncRNA-mRNA regulatory relationships were shared by the four cancers, suggesting that different cancers had varied ceRNA networks [23]. In leukemia, CML and AML ceRNA networks based on shared miRNAs and MREs were constructed. Results showed that 6 (CDKN1A, ABL1, BTN2A1, ENPP1, CNST and SYNM) and 2 (CLOCK and SUZ12) sub-ceRNA networks for CML and AML respectively [12]. However, the detail of lncRNA-miRNA-mRNA ceRNA network did not be presented in AML with prognosis.

In the present study, the significantly different expression levels of mRNAs in AML were calculated (Figure 1). Importantly, 120 overlapped genes were obtained from the predicted target mRNAs, WGCNA-turquoise-cyan mRNAs, as well as the significantly different up-regulated mRNAs and down-regulated mRNAs (Figure 2 and 3). To further investigate the relationships of these 120 genes with prognosis, univariate and multivariate cox proportional hazard regression analysis were applied. Then a survival model for 3-year OS with 8 genes: HOXA9+INSR+KRIT1+MYB+SPRY2+UBE2V1+WEE1+ZNF711, was set up (Figure 4). Finally, a ceRNA network was constructed by 108 lncRNAs, 10 miRNAs and 8 mRNAs (Figure 5), which could act as biomarkers based on the patients’ prognosis.

Among the 8 target genes, HOXA9, WEE1 and MYB had been demonstrated to be essential in leukemogenesis and disease process. HOXA9 had an important role in hematopoietic stem cell expansion, of which aberrant expression was a prominent feature of AML driven by diverse oncogenes. With continued study in HOXA9-mediated AML, there was a wealth of opportunity for developing novel therapeutics applicable for AML with HOXA9 overexpression [24]. MiR-182 was reported to regulate percentage of myeloid and erythroid cells in CML [25]. Thus, the relationship between HOXA9 and miR-182 needed to be investigated in AML as predicted. WEE1 kinase was crucial in the G2-M cell-cycle checkpoint arrest for DNA repair before mitotic entry. WEE1 was expressed at high levels in various cancer types including leukemia and was a validated target of the miR-17-92 cluster in leukemia [26], giving support to our prediction of miR-17-WEE1 axis in AML. MLL fusion proteins negatively regulated miR-150 production, and forced expression of miR-150 inhibited leukemic cell growth and delayed MLL-fusion-mediated leukemogenesis likely by targeting MYB, suggesting a miR-150-regulated MYB signaling underlying the pathogenesis of leukemia [27]. MiR-21 was considered to be an important miRNA, which was frequently elevated in all types of myeloid leukemia, while lncRNA MEG3 inhibited proliferation of CML cells by sponging MiR-21 [28]. Primary FLT3-ITD^+^ AML clinical samples had significantly higher miR-155 levels compared with FLT3 wild-type AML samples. MiR-155 collaborated with FLT3-ITD to promote myeloid cell expansion in vivo [29]. Besides, miR-106, miR-195, miR-424, miR-454 and miR-497 were all involved in the disease process of leukemia or solid tumors [30–34]. Therefore, many previous studies had given great experimental support to our prediction of the ceRNA network. Pivotally, Kaplan-Meier survival curves of our predicted model showed that patients with predicted high risk had significantly shorter OS time than those with low risk. Although the studies of lncRNAs in AML were limited, these predicted lncRNAs provided novel pathways or networks to study the function of 8-genes survival model in AML development and treatment.

This study defines ceRNA network from multiple dimensions, and provides possible prognostic markers for predicting patient outcome, which will help to increase our comprehension about ceRNA network-mediated leukemogenesis. Via this study, a novel perspective will be produced to make clear leukemia mechanisms and suggest approaches to regulate ceRNA networks for leukemia therapeutics.

## METHODS

### TCGA RNA sequence dataset

The RNA sequence data of 151 BMs with AML (Hematopoietic and reticuloendothelial systems) were retrieved from TCGA data repository (https://portal.gdc.cancer.gov/), which were derived from IlluminaHiSeq RNA-Seq platform. RNA-Seq data, miRNA-Seq and clinical data such as patient survival time and FAB classification information were obtained from TCGA.

### GTEx RNA sequence dataset

All data of normal tissue samples were obtained from 407 whole blood in GTEx V7 release version (https://gtexportal.org/home/datasets). Complete description of the donor genders, multiple ethnicity groups, wide age range, the biospecimen procurement methods and sample fixation were described in GTEx official annotation.

### Identification of differentially expressed genes

The ensemble ID of samples was converted by using GENCODE Gene Set-11.2017 version. LncRNAs and mRNAs ensemble ID that was not included in the GENCODE database were excluded.

R package (edgeR) was used to identify significant differentially expressed genes in AML and normal samples. All q values use FDR to correct the statistical significance of the multiple test. Absolute log2FC ≥2 and FDR < 0.05 were considered significant [35–37].

For the obtained differentially expressed mRNAs we generated volcano map using the ggplot2 packages in the R platform.

### Gene Ontology, Kyoto Encyclopedia of Genes and Genomes, and Gene Set Enrichment Analysis

ClusterProfiler was used for GO, KEGG and GSEA [38–40]. GO was used to describe gene functions along three aspects: biological process (BP), cellular component (CC) and molecular function (MF). The KEGG-GSEA was searched for pathways at the significance level set at p<0.05.

### Weighted correlation network analysis

WGCNA was an algorithm used in gene coexpression network identification by high-throughput expression profiles mRNAs or lncRNAs with different traits. Weighted coexpression relationship among all dataset subjects in an adjacency matrix was assessed using the pairwise Pearson correlation analysis. In this study, WGCNA was used to analyze mRNAs and lncRNAs to obtain the most related mRNAs or lncRNAs with AML patients.

### MiRNA regulatory network

MiRcode (http://www.mircode.org/) was used to predict interactions between lncRNAs and miRNAs. StarBase (http://starbase.sysu.edu.cn/), miRTarBase (http://mirtarbase.mbc.nctu.edu.tw/), miRDB (http://www.mirdb.org/) and TargetScan (http://www.targetscan.org/) databases were used to explore target mRNAs.

### Cox regression analysis

A univariate cox proportional hazards regression analysis was employed to identify the relationship between the expression level of mRNAs and patient’s OS. Thereafter, multivariate cox analysis was employed to evaluate the contribution of the selected genes. The analysis was conducted using the R package of survival.

## Supplementary Material

Supplementary Figure

Supplementary Table
