# Supplementary material for: Prediction of competing endogenous RNA coexpression network as prognostic markers in AML
Source: Aging (Albany NY). 2019 May 31;11(10):3333–47. doi: 10.18632/aging.101985 (PMC6555472; doi:10.18632/aging.101985)
Supplement: Supplementary Table [file aging-11-101985-s002.pdf]

SUPPLEMENTARY FIGURE

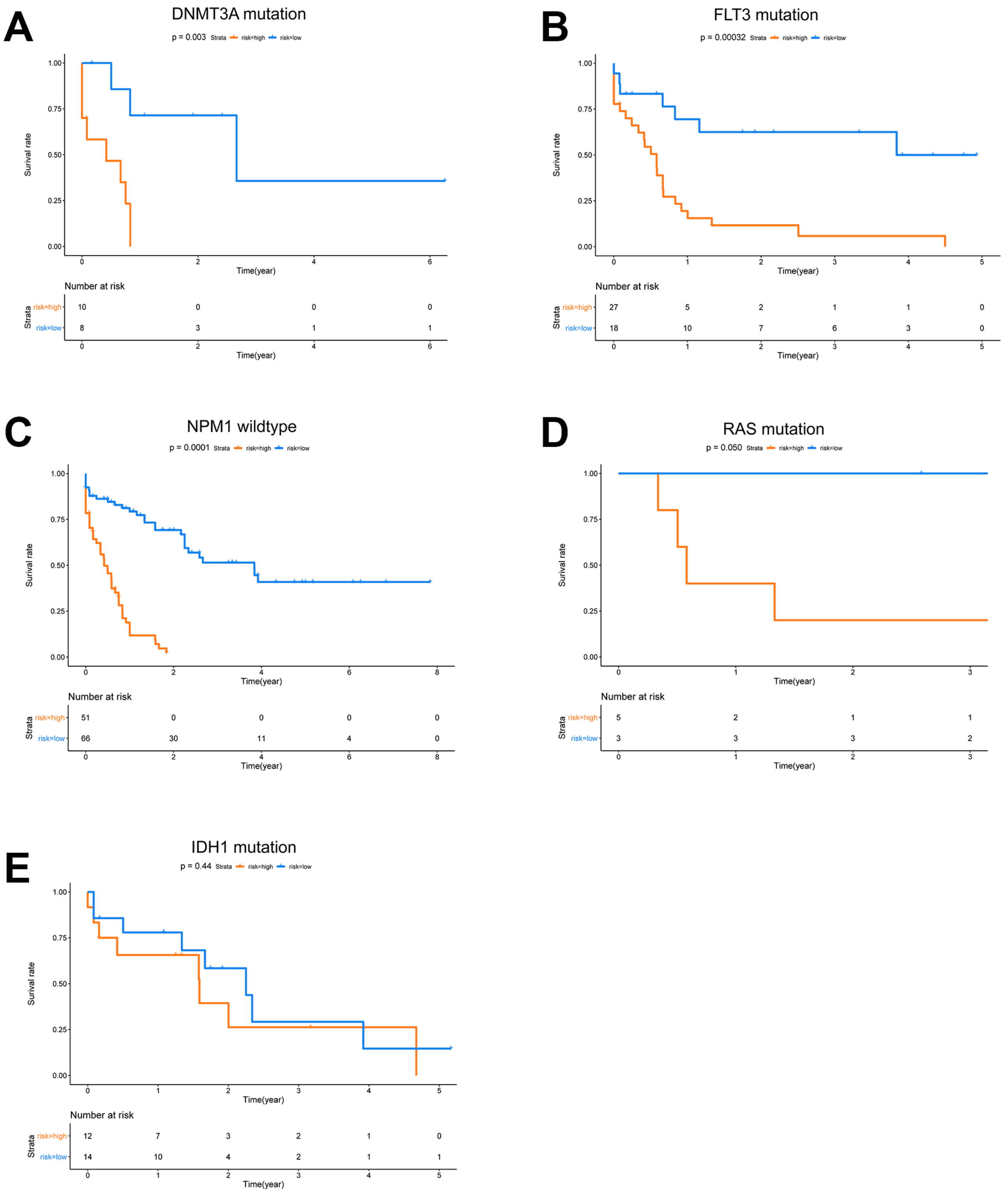

Supplementary Figure 1. Survival analysis of AML patients according to the gene mutation. Kaplan-Meier survival analysis of the 8 genes was performed in patients with DNMT3A mutation (**A**), FIT3 mutation (**B**), NPM1 wildtype (**C**), RAS mutation (**D**) and IDH1 mutation (**E**).
